# Supplementary material for: Does Shoot Age Influence Biological and Chemical Properties in Black Currant (Ribes nigrum L.) Cultivars?
Source: Plants (Basel). 2022 Mar 24;11(7):866. doi: 10.3390/plants11070866 (PMC9003360; doi:10.3390/plants11070866)
Supplement: Supplementary file 1 [file plants-11-00866-s001.zip › plants-1634451-supplementary.pdf]

Does shoot age influence biological and chemical properties in black currant (*Ribes nigrum* L.) cultivars?

Boban Djordjevic[1]*, Dejan Djurovic[1], Gordan Zec[1], Dragana Dabić Zagorac[2], Maja Natić[3], Mekjell Meland[4], Milica Fotirić Akšić[1]

[1]Faculty of Agriculture, University of Belgrade, Nemanjina 6, 11080 Belgrade, Serbia

[2]Innovation Centre, Faculty of Chemistry Ltd., Studentski trg 12-16, 11000 Belgrade, Serbia

[3]Faculty of Chemistry, University of Belgrade, P.O. Box 51, 11158 Belgrade, Serbia

[4]Norwegian Institute of Bioeconomy Research (NIBIO), Ullensvangvegen1005, N-5781 Lofthus, Norway

* Corresponding author: b.djordjevic@agrif.bg.ac.rs

Content: **Table S1.** Average data (2018/2109) for 13 studied black current cultivars studied

Table S1. Average data (2018/2109) of all analyzed traits in 13 black current cultivars studied.

No	Variables	Samples																									
		**Ben sarek**		**Ben nevis**		**Bona**		**Ben lomond**		**Ometa**		**Tenah**		**Silmu**		**Titania**		**Moling juel**		**Ojebin**		**Tsema**		**Triton**		**Čačanska crna**	
		**3 year**	**2 year**	**3 year**	**2 year**	**3 year**	**2 year**	**3 year**	**2 year**	**3 year**	**2 year**	**3 year**	**2 year**	**3 year**	**2 year**	**3 year**	**2 year**	**3 year**	**2 year**	**3 year**	**2 year**	**3 year**	**2 year**	**3 year**	**2 year**	**3 year**	**2 year**
		**BS 3‡**	**BS 2**	**BN 3**	**BN 2**	**BO 3**	**BO 2**	**BL 3**	**BL 2**	**O M3**	**O M2**	**TE 3**	**TE 2**	**SI3**	**SI2**	**TI3**	**TI2**	**MJ 3**	**MJ 2**	**OJ 3**	**OJ 2**	**TS 3**	**TS 2**	**TR 3**	**TR 2**	**CC 3**	**CC 2**
1†	Bud burst	59.50	62.50	61.00	63.00	56.50	58.50	61.50	65.50	60.50	64.00	61.00	63.50	60.50	64.00	67.50	73.50	59.00	63.50	60.00	63.50	60.00	65.00	59.50	62.00	60.00	63.00
2	Beginning of blooming	84.00	88.50	84.00	88.00	81.50	84.00	82.00	87.00	83.00	86.50	84.50	86.50	84.50	87.00	84.00	87.00	83.00	84.50	83.50	85.50	81.50	86.00	83.50	85.50	81.50	85.00
3	Full blooming	99.50	102.00	100.00	103.50	94.50	99.00	97.00	101.50	101.00	104.00	100.50	104.00	101.00	103.50	101.00	104.00	95.00	97.00	101.50	104.00	95.00	99.00	98.50	102.50	94.00	97.00
4	Beginning of harvest	167.50	172.00	173.50	178.00	160.00	165.00	176.50	179.00	182.00	187.50	172.00	175.00	169.00	173.50	170.00	174.00	175.00	179.00	175.00	179.00	172.00	177.50	170.00	173.50	172.00	175.50
5	Cluster weight (g)	9.65	7.45	7.35	5.40	12.35	8.80	7.25	5.80	7.10	5.80	9.30	7.80	7.90	6.60	10.30	7.80	9.10	7.30	7.00	5.90	11.80	8.60	7.30	6.05	9.05	7.35
6	Cluster length	5.70	5.45	5.35	4.90	5.50	4.45	7.00	6.60	6.35	5.70	6.45	5.95	5.70	5.95	6.25	5.60	7.70	6.65	5.35	5.30	8.50	7.70	6.20	5.75	8.50	7.50
7	Number of flowers per cluster	8.30	6.85	7.25	6.55	8.75	8.05	8.20	7.45	9.45	8.30	8.95	8.20	11.40	9.30	10.20	8.85	10.00	8.65	6.90	6.60	13.85	11.15	9.60	8.45	10.75	9.60
8	Number of berries per cluster	6.45	5.50	4.75	4.45	6.45	5.90	7.15	6.35	7.15	6.95	6.40	6.05	7.30	6.05	8.80	7.75	7.80	6.65	5.95	5.95	9.45	8.30	6.85	6.05	8.60	7.40
9	Fruit set	78.95	80.05	65.35	67.85	76.70	75.30	87.00	85.10	76.95	83.55	71.40	73.80	64.40	65.15	86.30	86.70	79.10	77.95	85.65	88.75	68.60	74.45	71.60	73.60	80.20	76.50
10	Berry weight (g)	1.60	1.25	1.65	1.20	2.05	1.45	1.05	0.90	1.05	0.80	1.50	1.20	1.05	1.05	1.15	1.00	1.20	1.10	1.25	1.00	1.15	1.05	1.15	0.95	1.10	0.95
11	Berry diameter (mm)	14.00	12.60	14.00	12.95	15.45	14.80	12.05	10.85	12.05	11.05	13.75	13.30	12.55	11.20	12.75	11.95	12.60	11.85	12.70	11.60	12.30	11.90	12.55	10.90	12.55	11.80
12	Length of shoot (cm)	64.40	94.80	78.50	114.75	72.15	110.40	107.15	146.25	79.50	125.95	66.65	114.45	96.20	146.20	81.00	135.95	98.60	153.15	69.10	110.40	101.95	130.25	58.35	119.55	80.65	126.70
13	Number of clusters per shoot	19.45	62.95	17.00	66.20	16.30	53.35	22.90	53.70	16.00	53.80	21.15	47.75	16.25	53.40	16.20	57.10	32.20	65.40	8.65	26.50	30.00	54.60	17.15	47.45	20.10	58.30
14	Yield per shoot (kg)	188.65	504.45	125.90	403.80	200.45	469.55	165.90	311.00	112.35	312.05	194.05	370.95	128.30	358.05	167.60	445.70	288.45	473.50	61.40	156.35	350.40	469.65	125.00	286.10	183.15	429.85
15	% of shoot yield in total yield	27.20	72.80	23.60	76.40	29.95	70.05	34.75	65.25	26.00	74.00	34.15	65.85	27.30	72.70	28.30	71.70	38.35	61.65	27.90	72.10	42.60	57.40	29.95	70.05	30.35	69.65
16	total phenols	212.30	177.80	189.60	169.30	172.30	154.25	221.95	194.95	250.30	229.75	160.60	148.45	157.25	138.75	151.25	138.25	205.30	182.25	166.25	139.80	189.10	162.35	171.30	152.05	211.90	193.85
17	total antoc aglicones	609.70	517.90	662.55	466.70	569.05	485.80	679.55	475.15	963.30	682.00	439.50	328.85	607.45	529.20	539.45	492.90	626.95	460.25	523.55	472.70	602.35	430.65	551.95	520.30	553.45	485.10
18	del 3 rutin	254.70	200.30	250.80	203.60	243.50	189.10	260.15	198.40	382.25	272.20	218.05	158.00	253.10	198.70	218.60	197.25	240.95	179.20	218.60	191.95	240.95	151.90	218.60	191.95	262.70	223.20
19	del 3 glu	115.25	85.50	112.30	98.15	104.05	78.05	136.90	91.15	196.20	103.75	45.50	26.10	113.65	87.65	99.15	73.15	136.90	106.15	93.85	67.85	124.60	93.85	108.05	82.05	88.75	63.75
20	cya 3 rut	221.95	218.90	255.25	134.35	210.75	207.70	234.50	164.70	317.15	241.90	152.95	127.70	220.35	222.30	205.85	207.80	215.30	145.50	200.55	202.50	203.00	155.50	200.55	221.70	170.00	172.80
21	cya 3 glu	17.80	13.20	44.20	30.60	10.75	10.95	48.00	20.90	67.70	64.15	23.00	17.05	20.35	20.55	15.85	14.70	33.80	29.40	10.55	10.40	33.80	29.40	24.75	24.60	32.00	25.35

2		3.5	4.2	3.6	4.1	4.1	4.4	2.8	3.1	3.2	3.5	4.4	4.8	3.8	4.2	5.0	5.6	3.7	4.1	5.0	5.3	3.3	3.6	2.6	2.7	3.5	3.8
2	dpph	0	0	0	5	0	0	5	5	5	5	0	0	0	5	5	0	0	0	5	0	5	5	0	5	5	5
2		2.0	1.8	2.2	1.9	1.2	1.1	1.3	1.2	1.5	1.0	1.7	1.2	1.1	1.0	1.1	0.8	1.4	1.3	1.0	0.8	1.2	1.2	1.0	0.9	0.9	0.8
3	kempfer	0	5	0	0	0	0	0	0	5	0	5	0	5	0	0	0	5	0	5	5	0	5	5	5	5	5
2		3.0	3.2	3.2	3.0	2.8	2.5	2.7	2.7	2.1	1.9	2.8	2.6	2.9	2.5	2.6	2.8	2.2	2.4	2.0	2.2	3.4	3.0	2.0	1.8	2.4	2.3
4	myricetin	5	0	0	0	0	0	0	0	0	0	5	5	0	5	0	5	0	5	0	5	5	5	0	5	5	0
2		7.1	6.2	7.3	5.8	7.3	6.5	5.4	5.1	8.3	7.2	8.1	7.0	5.8	5.5	4.1	3.8	7.3	7.0	6.4	5.3	8.2	7.6	5.2	5.3	7.2	7.2
5	quercetin	5	5	0	5	0	0	0	5	0	0	0	0	0	0	5	5	5	5	0	0	5	5	0	5	0	0
2		12.	11.	12.	10.	11.	10.	9.4	9.1	11.	10.	12.	10.	9.8	9.0	7.8	7.5	11.	10.	9.3	8.3	12.	11.	8.2	8.0	10.	10.
6	total flavanoids	20	25	70	75	25	10	0	0	95	10	65	80	5	5	5	0	00	80	5	5	90	95	5	5	50	35
2	Total soluble	12.	11.	12.	11.	11.	9.7	13.	11.	17.	14.	13.	11.	14.	12.	13.	10.	14.	11.	13.	10.	13.	11.	14.	12.	14.	11.
7	solids (%)	40	10	65	10	10	5	60	60	25	45	55	35	85	10	30	40	35	45	40	75	55	00	20	70	25	75
2		2.2	2.2	2.3	2.3	1.7	1.8	1.6	1.6	2.4	2.4	1.8	1.9	1.7	1.6	1.3	1.4	1.8	2.0	2.1	2.1	1.8	1.8	1.8	2.0	1.8	2.0
8	Total Acids (%)	5	0	0	0	5	0	0	5	0	0	5	0	0	5	5	5	0	5	5	0	0	5	0	0	0	0
2		8.8	8.0	9.0	7.1	8.7	7.0	8.3	7.7	13.	10.	9.4	8.3	10.	9.4	9.2	8.0	10.	8.7	9.0	7.4	9.4	7.6	10.	9.7	11.	8.1
9	Total sugars (%)	0	5	5	0	5	5	5	0	40	25	0	5	30	0	0	5	20	0	0	0	5	5	65	5	85	5
3		3.9	3.6	3.9	3.2	5.8	4.6	5.2	4.7	6.0	4.8	5.0	4.5	6.1	6.0	6.9	5.7	5.7	4.3	4.4	3.9	5.3	4.4	6.1	5.4	7.4	4.6
0	Sweetness	0	5	0	0	0	0	0	5	5	0	0	0	0	5	5	0	0	0	0	0	5	5	0	5	0	0
3	Ascorbic acid	125	134	149	113	130	113	142	136	137	123	132	126	135	119	133	123	149	153	115	100	158	123	140	113	152	153
1	(mg/100 g)	.00	.30	.55	.20	.45	.80	.00	.95	.90	.95	.90	.80	.00	.05	.35	.90	.00	.40	.75	.80	.40	.90	.90	.60	.75	.30

ǂ Abbreviations correspond to the score plots in PCA analysis (Fig 2A).

† Abbreviations correspond to the factor loadings in PCA analysis (Fig 2B).
